# Mitochondrial genotype alters the impact of rapamycin on the transcriptional response to nutrients in *Drosophila*

**DOI:** 10.1186/s12864-021-07516-2

**Published:** 2021-03-24

**Authors:** John C. Santiago, Joan M. Boylan, Faye A. Lemieux, Philip A. Gruppuso, Jennifer A. Sanders, David M. Rand

**Affiliations:** 1 Department of Molecular Biology, Cellular Biology and Biochemistry, Brown University, Providence, RI 02912 USA; 2 Department Pathology & Laboratory Medicine, Brown University, Providence, RI 02912 USA; 3 Department of Pediatrics, Rhode Island Hospital, Providence, RI 02903 USA; 4 Department of Ecology and Evolutionary Biology, Brown University, Providence, RI 02912 USA

**Keywords:** Mitochondrial introgression, Mitonuclear genotype, Rapamycin, mTORC1

## Abstract

**Background:**

In addition to their well characterized role in cellular energy production, new evidence has revealed the involvement of mitochondria in diverse signaling pathways that regulate a broad array of cellular functions. The mitochondrial genome (mtDNA) encodes essential components of the oxidative phosphorylation (OXPHOS) pathway whose expression must be coordinated with the components transcribed from the nuclear genome. Mitochondrial dysfunction is associated with disorders including cancer and neurodegenerative diseases, yet the role of the complex interactions between the mitochondrial and nuclear genomes are poorly understood.

**Results:**

Using a *Drosophila* model in which alternative mtDNAs are present on a common nuclear background, we studied the effects of this altered mitonuclear communication on the transcriptomic response to altered nutrient status. Adult flies with the ‘native’ and ‘disrupted’ genotypes were re-fed following brief starvation, with or without exposure to rapamycin, the cognate inhibitor of the nutrient-sensing target of rapamycin (TOR). RNAseq showed that alternative mtDNA genotypes affect the temporal transcriptional response to nutrients in a rapamycin-dependent manner. Pathways most greatly affected were OXPHOS, protein metabolism and fatty acid metabolism. A distinct set of testis-specific genes was also differentially regulated in the experiment.

**Conclusions:**

Many of the differentially expressed genes between alternative mitonuclear genotypes have no direct interaction with mtDNA gene products, suggesting that the mtDNA genotype contributes to retrograde signaling from mitochondria to the nucleus. The interaction of mitochondrial genotype (mtDNA) with rapamycin treatment identifies new links between mitochondria and the nutrient-sensing mTORC1 (mechanistic target of rapamycin complex 1) signaling pathway.

**Supplementary Information:**

The online version contains supplementary material available at 10.1186/s12864-021-07516-2.

## Background

Mitochondria are specialized energy producing organelles known for their role in eukaryotic cellular energy production through oxidative phosphorylation (OXPHOS). Regulation of this essential process has an additional level of complexity relative to other cellular functions in that the components of the respiratory chain are encoded by two genomes, the nuclear genome and the mitochondrial genome (mtDNA). Four of the five OXPHOS complexes have components encoded by the mtDNA. These 13 complex subunits are the only protein coding genes in the mitochondrial genome with the remaining ~ 1200 proteins of the mitochondrial proteome encoded by the nuclear genome [1]. This results in a system that requires coordinated gene and protein expression between the two genomes to regulate mitochondrial function. Mitochondrial and nuclear genomes from the same population or species co-evolved due to shared inheritance [2]. When mtDNA from a distinct population or species is placed in a ‘foreign’ nuclear genetic background, coordinated functions may be disrupted resulting in unfavorable epistatic interactions. The extent to which such negative ‘mitonuclear interactions’ could impact natural metabolic signaling is not well characterized.

Mitochondrial functional capacity is closely monitored and regulated through a network of mitonuclear communication signals. Retrograde signals are those generated by the mitochondria, and anterograde signals are those generated by the nucleus and other organelles to regulate mitochondrial function. Since mitochondria play such a critical role in cellular homeostasis, any deficiencies in this mitonuclear communication network become particularly relevant during times of limited nutrient availability. Nutrients need to be readily available for metabolism at all times in order to provide a constant supply of substrates for the OXPHOS pathway, regardless of organismal nutrient intake levels. In situations where nutrient intake is not sufficient to fuel glycolysis, cellular signaling can promote utilization of fatty acids and amino acids as alternative energy sources. This function requires efficient and coordinated responsiveness to changes in nutrient availability in order to shift metabolite utilization.

An integral component of the metabolic homeostasis signaling network is the target of rapamycin (TOR) kinase. When functioning in the heteromeric protein complex mTORC1, it regulates autophagy, cellular growth and proliferation through a diverse array of functional pathways [3]. In regulating these functions to meet cellular needs, mTORC1 is inherently integrated into the network of mitonuclear communication. Studies using the mTOR specific inhibitor rapamycin have demonstrated the role of mTORC1 in mitochondrial anterograde signaling. These anterograde signaling effects include mediating mitochondrial function, mitochondrial respiration, ROS production, mitophagy, mitochondrial morphology and mitochondrial biogenesis [4–10]. Conversely, retrograde signals generated by mitochondria have been shown to regulate mTORC1 activity. Mitochondrial retrograde signaling has been defined as the cellular response to changes in the functional state of mitochondria [11]. These include changes in AMP:ATP levels through AMP kinase, cytosolic calcium levels through calmodulin-dependent protein kinase kinase-β (CaMKK2), and mitochondrially generated reactive oxygen species (ROS) [12–19]. The diversity of metabolites that monitor and modify mitochondrial functional reflects the complexity of the metabolic regulation associated with the growth promoting function of mTORC1 while maintaining metabolic homeostasis.

Our study was designed to test the hypothesis that mitonuclear genotype impacts the cell’s capacity to respond to metabolic stress. To test this, we utilized a *Drosophila* mitochondrial introgression strain that has an mtDNA genotype from the species *D. simulans* (sm21 mtDNA haplotype) and a nuclear genome from the *D. melanogaster* line Oregon R. The generation of this introgression line was made possible by the unusual ability of female *D. simulans* C167.4 to produce progeny with male *D. melanogaster* [20]. The progeny of these mating events were then extensively backcrossed to achieve an isogenic *D. melanogaster* Oregon R nuclear genome carrying the *D. simulans* sm21 mtDNA [21, 22]. Since our mitochondrial introgression strain has mtDNA from one species and a nuclear genome from another, we use it as a model for a disrupted mitonuclear genetic interaction relative to a *D. melanogaster Oregon R* strain carrying its own native mtDNA. We examined the transcriptomic response to re-feeding in eviscerated abdomen samples from these lines over several time points, with and without exposure of the flies to rapamycin. Our aim was to determine if mitonuclear interactions alter the response to nutrient flux in a TOR-dependent manner. Our results show that alternative mitonuclear genotypes have a significant impact on the transcriptional responsiveness to re-feeding post starvation that is exaggerated with rapamycin treatment.

## Results

### Mitochondrial introgression alters the Transcriptomic response to Rapamycin during Refeeding

**Fig. 1:**
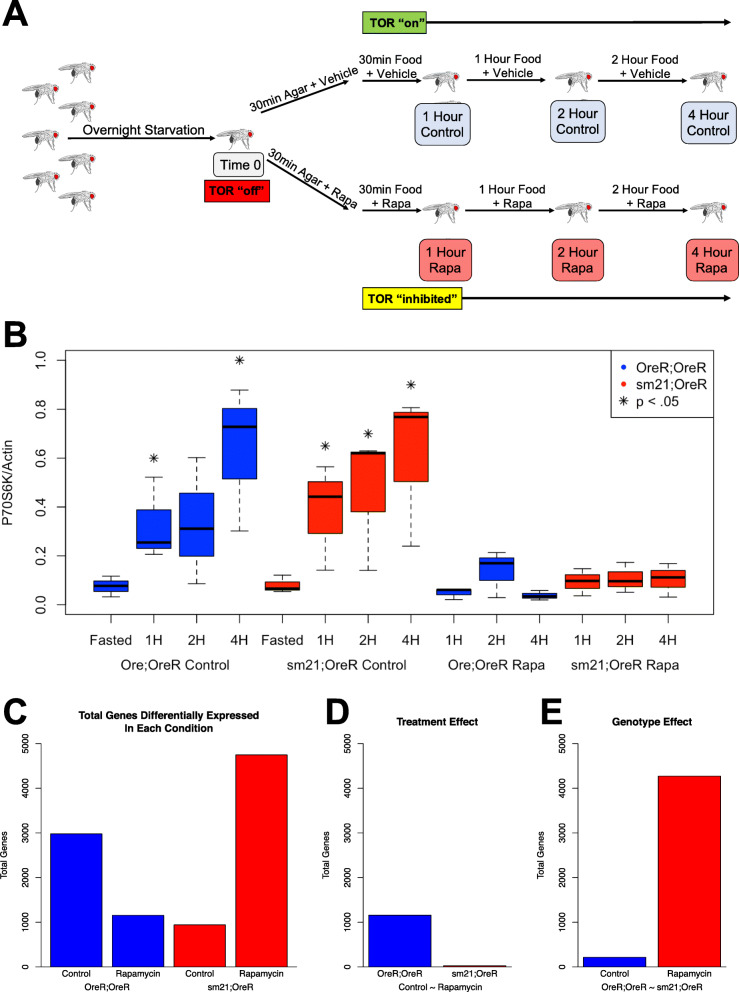
Time course transcriptome analysis evaluating the effect of mitochondrial introgression on the transcriptional response to rapamycin during refeeding. **a** Male flies were fasted for 12 h followed by treatment for 30 min with 200uM rapamycin or ethanol control on agar followed by refeeding with regular lab food containing 200uM rapamycin or ethanol. Samples were collected for transcriptome analysis at 4 time points including 0 (12 h fasting), 1 (30 min agar + treatment followed by 30 min food + treatment), 2 and 4 h post starvation. **b** Western blot analysis of total phosphorylated-P70S6K1 for OreR;OreR (red) and sm21;OreR (blue) flies in response to fasting (left), refeeding with control diet (center) or refeeding with food containing 200uM Rapamycin (right). The analysis was performed on whole fly samples in triplicate and the levels were normalized to total actin. Significant differences between the levels found in treated samples and fasted samples were determined using an unpaired t-test *p*-value cutoff of 0.05 (* = *p* < 0.05). **c** Total genes detected by ImpulseDE2 that show a significant response pattern to refeeding over the full 4 h time course within each GxT condition. Genotype by treatment time course conditions from left to right: OreR;OreR control (left blue); OreR;OreR rapamycin (right blue); sm21;OreR control (left red); sm21;OreR rapamycin (right red). **d** Total genes detected by ImpulseDE2 that show a significantly different response pattern to refeeding with and without rapamycin treatment over the full 4 h time course within a mitonuclear genotype. Left/blue: The total number of genes with a significant difference between the OreR;OreR control and OreR;OreR rapamycin treated time courses. Right/red: The total number of genes with a significant difference between the sm21;OreR control and sm21;OreR rapamycin treated time courses. **e** Total genes detected by ImpulseDE2 that show a significantly different response pattern between mitonuclear genotypes over the full 4 h time course within control or rapamycin treated conditions. Right/red: The total number of genes with a significant difference between the OreR;OreR rapamycin treated and sm21;OreR rapamycin treated time courses. For all data, a Benjamini-Hochberg FDR adjusted *p*-value (adj. *p*-value < 0.05) was used for determining significant differential gene expression

In order to examine the effect of altered mitonuclear genetic interactions on metabolic stress response pathways, we performed a time course transcriptome analysis on two *Drosophila* mitonuclear genotypes (raw reads are publicly available from the NCBI Sequence Read Archive (SRA) (https://www.ncbi.nlm.nih.gov/sra) under BioProject accession: PRJNA610872 and the aligned gene read count table is available as Supplementary Table S1). We studied four time points starting from a starved state and ending after 4 hours of refeeding with or without rapamycin treatment (Fig. 1a). Conducting the experiment across these short treatment times was critical for addressing the innate responsiveness of each genotype to significant shifts in nutrient availability. Since our focus is on the interaction between mitonuclear genetic interactions and mTORC1 signaling networks, we performed a western blot analysis to detect levels of phosphorylated ribosomal protein S6 kinase-1 (phospho-P70S6K1) at each timepoint and treatment in both genotypes (Fig. 1b and Supplementary Figure S1). Increased levels of phospho-P70S6K1 are an indicator of increased mTORC1 activity that is inhibited by treatment with Rapamycin [23–25]. This analysis shows increased mTORC1 activity in flies refed with the control diet, but not in flies refed with Rapamycin treatment, when compared to those from the fasted state. The increase in mTORC1 activity was observed within the first hour of treatment, demonstrating that both the refeeding and drug are inducing an effect within the first hour suggesting that gene expression could be changing in a similar time frame. Notably, mTORC1 activity is distinctly increased in response to refeeding after fasting compared to the fed state indicating a critical role of mTORC1 in this metabolic stress state (Supplementary Figure S1).

Because gene expression differences across such a short time course and treatment time could be difficult to detect in the whole fly, we decided to focus our analysis on a subset of tissues to increase the concentration of significant regulatory effects. We chose to measure expression in the eviscerated abdomen which, in *Drosophila*, is where many of the tissues responsible for maintaining metabolic homeostasis are located including the fat body, heart and muscle tissue [26]. The transcriptome analysis was done on male eviscerated abdomens from the “home team” line (OreR;OreR; *D. melanogaster Oregon R* mtDNA and nuclear genome, following the notation: mtDNA;nuclearDNA) and the mitochondrial introgression “away team” line (sm21;OreR; *D. simulans sm21* mtDNA and *D. melanogaster Oregon R* nuclear genome). Males were chosen over females to limit variation in nutrient stress response since it has been demonstrated that mating status and egg production can have a significant impact on nutrient intake that is in part mediated by mTOR signaling [27–29]. Since the two genotypes have isogenic nuclear genomes but different mitochondrial genomes (mtDNA), any differences in the transcriptional response to refeeding and rapamycin treatment between the two lines can be attributed to the presence of a non-native mitonuclear genetic interaction.

The transcriptome analysis was performed specifically to take advantage of our time course model while capturing the responsive elements to refeeding and rapamycin. Individual time points were tested for genes with significant differential expression within their respective genotype by treatment (GxT) combination (four combinations of two alternative mtDNAs x rapamycin or control food treatments) using the R package EdgeR (Supplementary Table S2 A-L) [30]. A direct comparison between the two genotypes in the fasted state found that there are no significantly differentially expressed genes between the two genotypes suggesting that they are similarly affected. Alternatively, to determine the general effect of treatment at different timepoints, individual time points were tested relative to the starved state for each GxT combination. Volcano plots (Supplementary Figure S2 A-D) show the direction and magnitude of significantly differentially expressed genes at individual time points. Interestingly, each individual time point comparison had a distinct response pattern with no two GxT comparisons having similar effects of re-feeding. This is consistent with the presence of a transcriptional impact of rapamycin treatment and also of the mtDNA genotype on the overall response to re-feeding.

The time course design allowed us to detect variation between samples at any given time point, with each comparison addressing a distinct expression pattern between two conditions. By comparing individual treatment times between genotypes (Supplementary Figure S3), we see there is a transient difference in response to 2 h of refeeding with control food, but the response is observed by both genotypes at the next time point. However, in response to refeeding with rapamycin there is a sustained difference between genotypes that reflects the treatment response observed in sm21;OreR, but not OreR;OreR, at the early time points. These pairwise comparisons suggest a dynamic transcriptional response over time, but the volcano plots in Figure S2 make it difficult to demonstrate the nature of the transcriptional responses of the GxT effects across the multiple time points. The differences in expression levels between the 1 h and 4 h refeeding timepoints were validated using qPCR on samples prepared in an independent repeat experiment as described in the methods. To characterize the temporal aspects of the data, we utilized the R package ImpulseDE2 [31]. This program was designed specifically for the analysis of longitudinal data sets. It enabled us to test for genes whose expression changed significantly across time points within a time course, instead of merging data from analyses of individual time points. Using this method, we first examined the individual time course for each GxT combination to find genes that significantly changed in response to the re-feeding treatment (Fig. 1c, Supplementary Table S3). We then compared different pairs of GxT conditions for significant variation across time to test for effects of mtDNA genotype and rapamycin treatment in response to metabolic stress. In OreR;OreR, the total number of time-responsive genes was appreciably reduced with rapamycin treatment. This corresponded with the results of the analysis of individual time points (Fig. 1c left vs Supplementary Figure S2 A-B). Interestingly, the sm21;OreR genotype showed the opposite effect of rapamycin treatment, with fewer genes differentially expressed under the control diet than the treated diet. Note that when ImpulseDE2 detects significant differential expression in response to treatment for a gene, it does not indicate an increase or decrease in expression since it is incorporating multiple time points. Instead, it indicates that there is a significant shift in expression pattern across the time course.

To test for an impact of genotype on the transcriptional response to both refeeding and rapamycin, we compared the longitudinal data between two genotype or treatment conditions using ImpulseDE2. Instead of testing if a gene responded significantly to treatment over time relative to no change in a single time course, this approach identified genes whose response to refeeding differed between two time courses distinguished by a single factor. We began by looking at the effect of rapamycin treatment within a genotype by comparing the response within a genotype to refeeding with control food to the response to refeeding with rapamycin-containing food. Our analysis revealed that there were many more genes with different responses to rapamycin treatment in the OreR;OreR genotype than in the sm21;OreR genotype, indicating a greater impact of rapamycin treatment on the transcriptional response to refeeding in the “home team” line than in the “away team” line (Fig. 1d). We next examined the effect of mtDNA genotype by comparing the response in OreR;OreR samples to the response in sm21;OreR samples within a single treatment. While there were very few genes that responded differently between the two genotypes when refeeding with control food, there were over 4000 genes with a significantly different response to refeeding with rapamycin (Fig. 1e). The different results from pairwise comparisons in edgeR vs. time course comparisons in ImpulseDE highlight the importance of the distinct dynamics of each transcriptional response for the mitonuclear genotypes and rapamycin treatment. It is important to note that the magnitude of transcriptional changes in the time course can be small in terms of fold-change, but the significance comes from the difference from a flat-line of no temporal response. This distinction contributes to the different patterns observed in volcano plots compared to ImpuleDE2 analyses. Together these data suggest that mtDNA genotype alone does not have a notable impact on the transcriptional response to refeeding post starvation under control conditions, but it distinctly alters the response to refeeding in flies that were exposed to rapamycin.

### Mitonuclear genotype induces distinct expression profiles for genes in Core metabolic pathways in response to metabolic stress

**Fig. 2:**
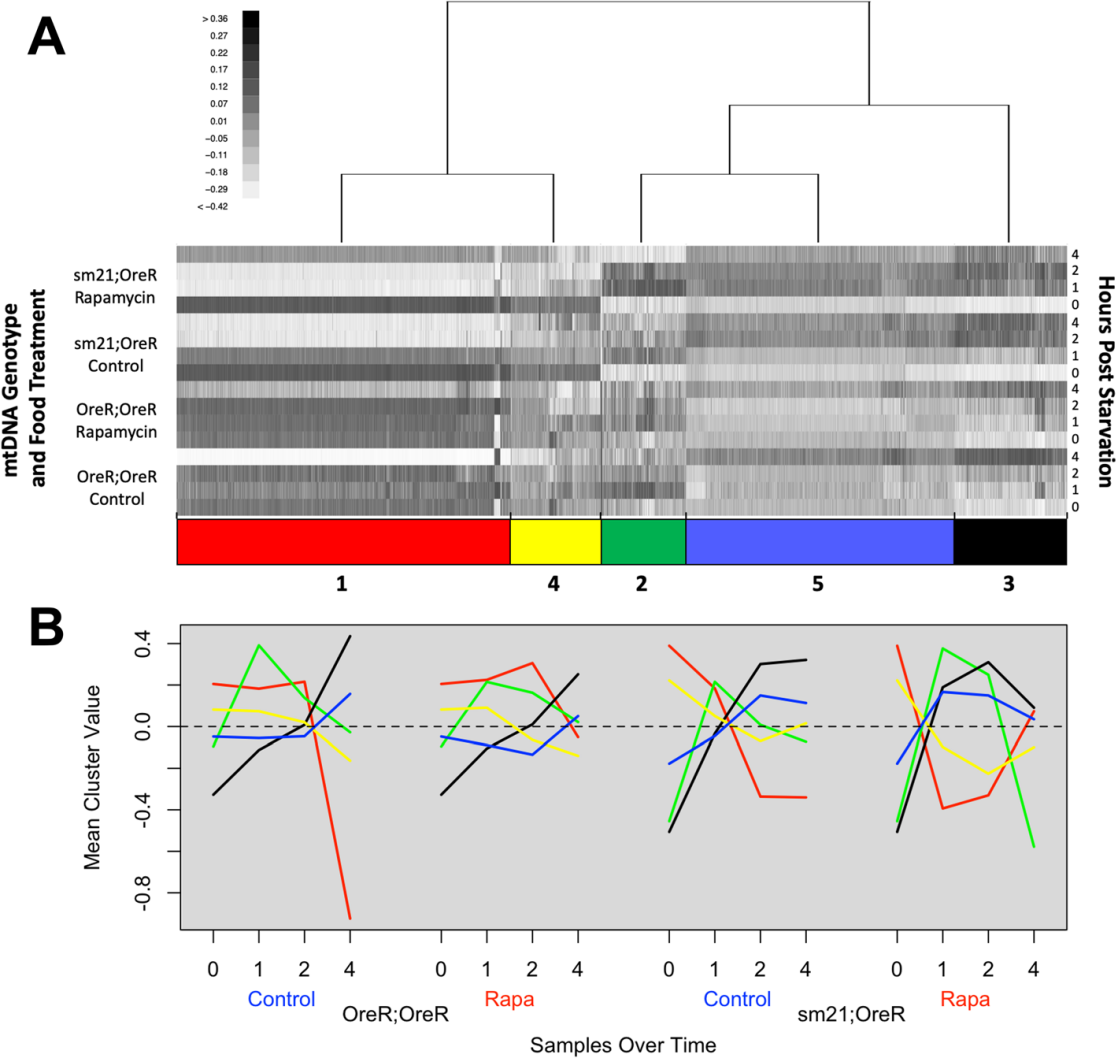
Model based clustering of time course expression profiles for differentially expressed genes. **a** All genes found to have significantly different response patterns by ImpulseDE2 in any of the different comparative analyses were clustered using the R package MBCluster-seq. The clustering is organized in the heatmap such that the rows are the individual conditions at each time point and the columns are the individual genes. The rows are grouped by Genotype x Treatment (GxT) condition ordered from 0 h (bottom) to 4 h (top) of refeeding after starvation. The grayscale of each column is the log-fold change of the normalized expression data standardized to the zero sum mean for a gene. Each expression pattern cluster has been associated with a given color and number for reference. **b** Mean values are plotted for expression across all genes within each cluster at each time point. Line color is used to identify the represented cluster

Having characterized genes with significantly different temporal patterns of expression between genotypes and treatments, we sought to identify clusters of genes with similar expression patterns that could help infer the functional significance of the transcriptional changes. To do this, we utilized the model based clustering R package MBCluster.seq [32] to perform expression profile clustering on the subset of genes determined to have a significant temporal response pattern by ImpulseDE2 in any of the conditions. The genes were stratified broadly into five expression clusters to observe general expression trends across large groups of genes (see methods for details on clustering, Supplementary Table S4). The clusters were organized in a heatmap (Fig. 2a) where the rows are each of the time points in a GxT condition and the columns are the individual genes. The rows are partitioned by GxT condition such that the four time points are sequential with starved state at the bottom and 4 h post starvation at the top. The columns are partitioned by cluster, and each cluster is manually assigned a color code for referencing the distinct expression profile in the remaining analyses. The mean data for each row within a cluster was plotted to visualize the general expression trend of the genes across the four condition time courses (Fig. 2b).

The resulting analysis showed distinct differences in mean expression profiles across genotypes and rapamycin treatments within a cluster (note reversal of ‘red’ cluster in sm21;OreR genotype under rapamycin). We interpreted this as indicating that the genes determined to have a significantly different response to a treatment or genotype condition could share common regulatory elements that are being differentially affected.

### Mitonuclear genetic interactions Alter the expression of genes in Core metabolic pathways

We next performed treatment-specific pathway enrichment analysis using the KEGG (Kyoto Encyclopedia of Genes and Genomes) pathway database (Supplementary Table S5). We did so in order to investigate the function of genes with genotype mediated differential expression [33–35]. As a baseline response, we analyzed KEGG pathway enrichment for the 2987 genes with a significant time course response for the “home team” OreR;OreR mitonuclear genotype in response to refeeding with control food (Fig. 1c). These genes were enriched for functional categories associated with mTORC1 signaling including purine metabolism, protein processing in endoplasmic reticulum, glycolysis, phagosome, pyruvate metabolism, longevity regulating pathway, and the citrate cycle. To determine the functional enrichment of genes with significantly different expression profiles between genotypes, we performed KEGG pathway enrichment analysis on the 215 genes found to have significant differential expression patterns between OreR;OreR and sm21;OreR in response to refeeding without rapamycin, and also on the 4271 genes with significant differential expression between genotypes in response to refeeding with rapamycin treatment (see Fig. 1e). We observed a complete absence of KEGG pathway enrichment for the control treatment genes. In contrast, the rapamycin treatment analysis detected 22 significantly enriched KEGG pathways with the most statistically significant being OXPHOS (Table 1). These pathways encompassed core metabolic functions involved in utilization of a diverse group of substrates. Interestingly, there were few genes found in these KEGG categories that had significant genotype-mediated differential expression in response to refeeding without rapamycin, implying that rapamycin enhances the transcriptional effect of the alternative mtDNAs on specific metabolic pathways.

**Table 1:** KEGG categories that are significantly enriched in mitonuclear response genes

KEGG Category	OreR;OreR vs sm21;OreR Rapa			OreR;OreR vs sm21;OreR Control		
	DE in Cat.	All in Cat.	adj *p*-value	DE in Cat.	All in Cat.	adj *p*-value
Oxidative phosphorylation	104	127	4.79E-22	0	127	1.00E+ 00
Proteasome	43	52	4.50E-09	0	52	1.00E+ 00
Glycolysis / Gluconeogenesis	42	54	7.00E-08	0	54	1.00E+ 00
Citrate cycle (TCA cycle)	31	41	1.62E-05	0	41	1.00E+ 00
Valine, leucine and isoleucine degradation	26	33	4.70E-05	0	33	1.00E+ 00
Fatty acid degradation	25	33	1.73E-04	0	33	1.00E+ 00
Pentose phosphate pathway	19	23	1.83E-04	0	23	1.00E+ 00
Galactose metabolism	27	37	3.15E-04	0	37	1.00E+ 00
Propanoate metabolism	22	29	4.35E-04	0	29	1.00E+ 00
Starch and sucrose metabolism	24	33	5.04E-04	2	33	1.00E+ 00
Phagosome	49	83	5.47E-04	2	83	1.00E+ 00
Fatty acid biosynthesis	12	13	7.52E-04	0	13	1.00E+ 00
Pyruvate metabolism	29	43	8.11E-04	0	43	1.00E+ 00
Glyoxylate and dicarboxylate metabolism	23	33	1.42E-03	0	33	1.00E+ 00
Peroxisome	49	85	1.64E-03	0	85	1.00E+ 00
Fructose and mannose metabolism	21	29	1.96E-03	0	29	1.00E+ 00
Protein processing in endoplasmic reticulum	68	131	2.89E-03	3	131	1.00E+ 00
Mitophagy	29	47	5.66E-03	1	47	1.00E+ 00
Vitamin B6 metabolism	6	6	2.23E-02	0	6	1.00E+ 00
Amino sugar and nucleotide sugar metabolism	27	47	2.63E-02	0	47	1.00E+ 00
Longevity regulating pathway - multiple species	27	51	2.66E-02	0	51	1.00E+ 00

The R package GOseq [36] was used to test for the enrichment of KEGG categories among the sets of genes found to have a significantly different response to refeeding with (left column) or without (right column) rapamycin between the OreR;OreR and sm21:OreR genotypes. The rows are the KEGG pathways found to be significantly enriched among the genes differentially expressed between genotypes in response to refeeding with rapamycin. The table sub-columns indicate as follows: “DE in Cat.” is the total number of significant responsive genes detected by ImpulseDE2 in that category; “All in Cat.” is the number of genes in the category that were used in the GOseq test; and the “adj. *p*-value” is the Benjamini-Hochberg corrected p-value for significant over representation in the category. A Benjamini-Hochberg FDR adjusted p-value (adj. *p*-value < 0.05) was used for determining significant KEGG pathway enrichment.

**Fig. 3:**
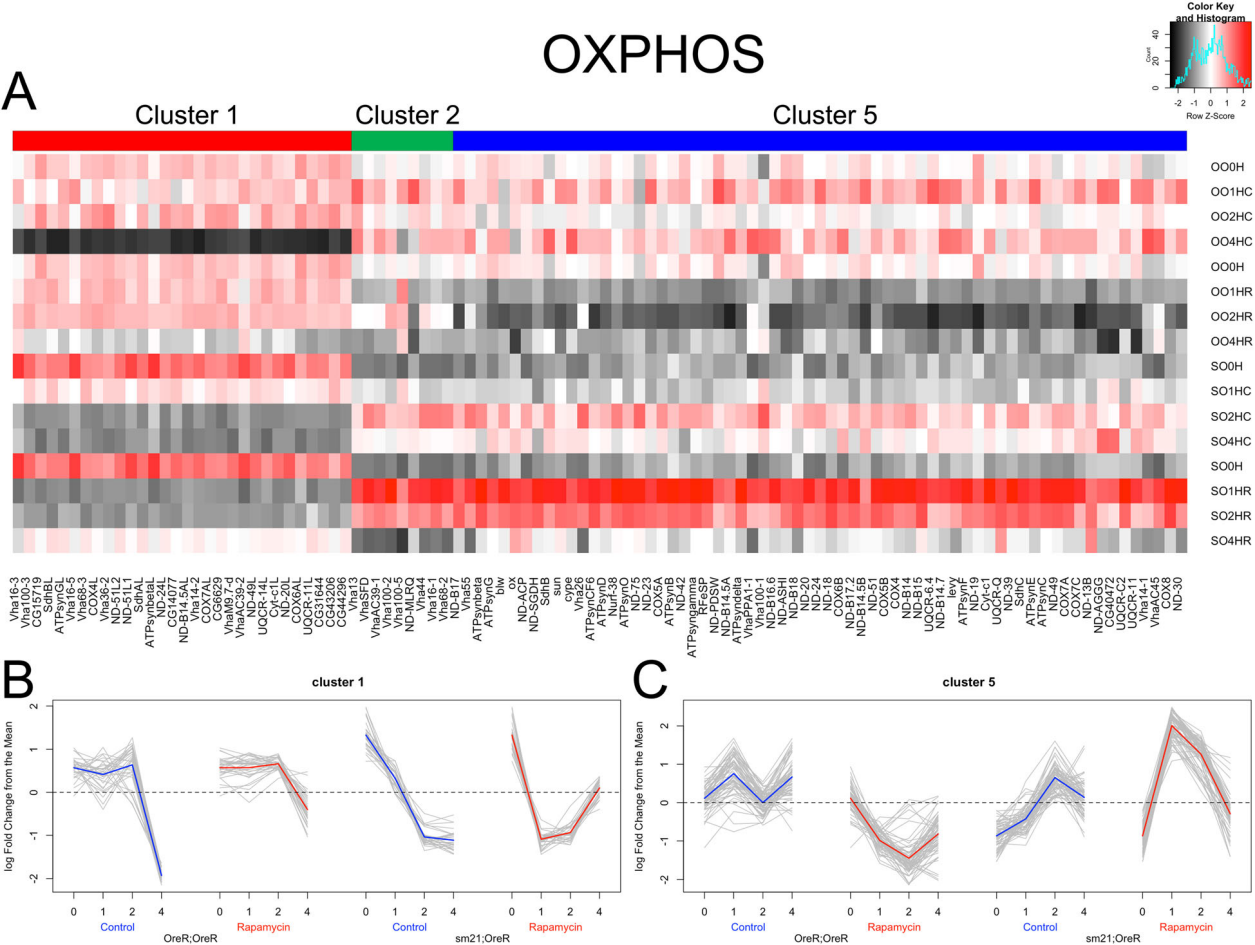
Mitonuclear genotype alters the transcriptional response of rapamycin sensitive OXPHOS genes. **a** A heatmap of the genes found to have significantly different response patterns to refeeding with rapamycin treatment between the OreR;OreR (OO) and sm21;OreR (SO) mtDNA genotypes. On the x-axis, the genes are organized and color coded according to the expression profiles established by MBCluster-seq while on the y-axis samples are separated into the four GxT condition time courses. The mean expression data is normalized by row, with the cell color indicating the z-score for the gene at a specified GxT time point. **b**-**c** Normalized expression data for the OXPHOS genes in cluster 1 (**b**) and cluster 5 (**c**) plotted as grey lines with the cluster means overlaid in blue for control treated samples and red for rapamycin treated samples

To understand how these pathways were being differentially regulated by the two genotypes in response to refeeding and rapamycin, we analyzed the expression patterns of the genes enriched in each KEGG category. Expression data for KEGG pathway-specific gene sets were stratified by their associated expression profile cluster generated by MBCluster-seq (Fig. 2) and then plotted as heatmaps to observe relative shifts in expression (Fig. 3a and Supplementary Figure S4). The majority of genes in 15 of the 22 enriched KEGG categories were primarily represented by expression profile clusters 1 and 5, as can be seen for OXPHOS, the most significantly enriched pathway (Fig. 3a). While the genes in these clusters both contributed to the same KEGG pathway, they showed distinctly different expression profiles for the rapamycin treated samples. Specifically, these expression clusters showed two instances of inverse directionality that have particularly significant implications when interpreting the data. First, this inverse dynamic was observed in cluster 1 (Fig. 3b) and also in cluster 5 (Fig. 3c), where changes in transcript levels for OreR;OreR during the response to rapamycin were opposite the changes observed in the sm21;OreR rapamycin treated samples. For both of these expression profiles, the most drastic difference in total gene expression was observed as a transient shift in the 1 and 2 h post-starvation time points. This instance of an opposite transient response suggested that the short-term response to rapamycin was altered by mitonuclear genotype in both sets of genes. Second, there was an inverse relationship between the expression profiles of rapamycin treated samples between the two clusters. This opposite transcriptional response was detected when comparing the gene expression profiles for OreR;OreR or sm21;OreR treated with rapamycin in cluster 1 (Fig. 3b) to the expression profile of genes from the same sample in cluster 5 (Fig. 3c). This was again an instance of opposite transient response, but this relationship implies that the gene sets themselves were responding oppositely to rapamycin treatment in both mitonuclear genotypes. Taken together, these two clusters represented gene sets that were differentially responsive to rapamycin treatment and mitonuclear genotype.

### Functional roles for genes in opposing expression profile clusters have overlapping nodes in KEGG pathways

**Fig. 4:**
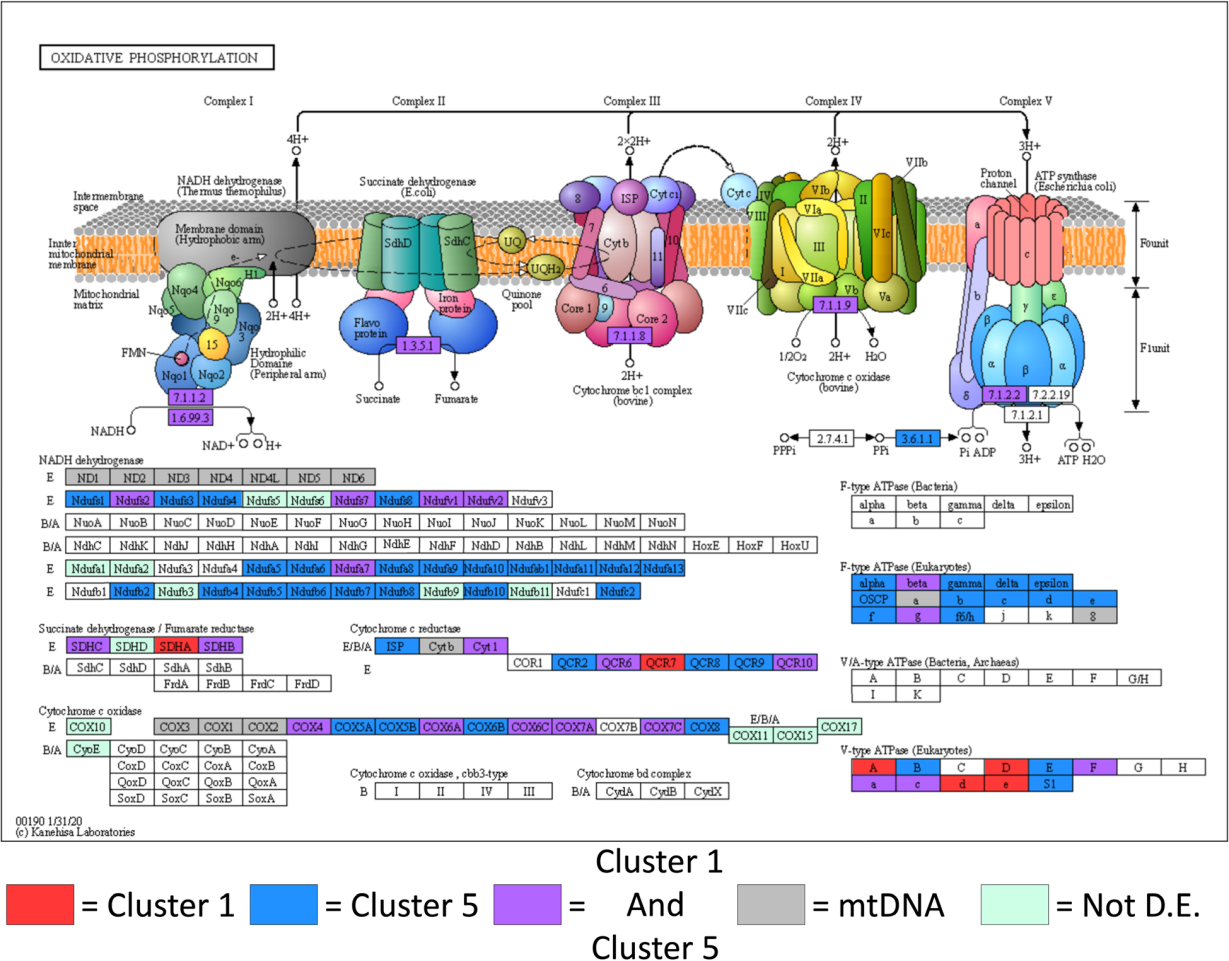
Functional mapping of opposing expression profiles in OXPHOS genes. The functional roles of OXPHOS genes with mitonuclear genotype mediated significant differential expression in response to refeeding with rapamycin. Genes were visualized in the OXPHOS metabolic pathway using the Kaneisha Laboratories online tool KEGG mapper (https://www.kegg.jp/kegg/tool/map_pathway2.html) [37]. KEGG pathway nodes are color-coded to correspond with their expression profile. Red nodes indicate that only genes from the “cluster 1” expression profile were associated, blue nodes indicate only “cluster 5”, purple nodes indicate both “cluster 1” and “cluster 5”, grey nodes indicate genes encoded by the mtDNA, green nodes indicate the associated genes were not differentially expressed and white nodes indicate there are no associated *D. melanogaster* genes. Copyright permission for image publication was obtained from Kanehisa Laboratories for KEGG pathway map ID 00190 “Oxidative Phosphorylation” copyright Kanehisa Laboratories [33–35, 37]

In order to determine if the observed opposing expression profiles within KEGG categories were associated with distinct functional roles, we mapped the genes to their enriched functional pathway using KEGG mapper [37] (Fig. 4). By coloring the nodes of the KEGG pathway diagram to correlate with the expression profile cluster, this mapping allowed us to visualize the connection between altered transcriptional response patterns and position in the network of biochemical pathways. Examination of the OXPHOS pathway revealed that the genes associated with each expression profile cluster were not segregated into distinct functional roles. Instead, genes from each cluster were found distributed throughout each of the five OXPHOS enzyme complexes (Fig. 4). Additionally, several nodes were found to have associated genes from both cluster 1 and cluster 5 due to mapping of functional isoforms such as gene duplications. This suggested that even though the OXPHOS genes associated with cluster 1 and cluster 5 have opposite transcriptional patterns in response to rapamycin, they code for products with similar functional roles.

### Co-expressed Rapamycin sensitive genes in clusters with opposite expression profiles are associated with male specific transcriptional regulation

**Fig. 5:**
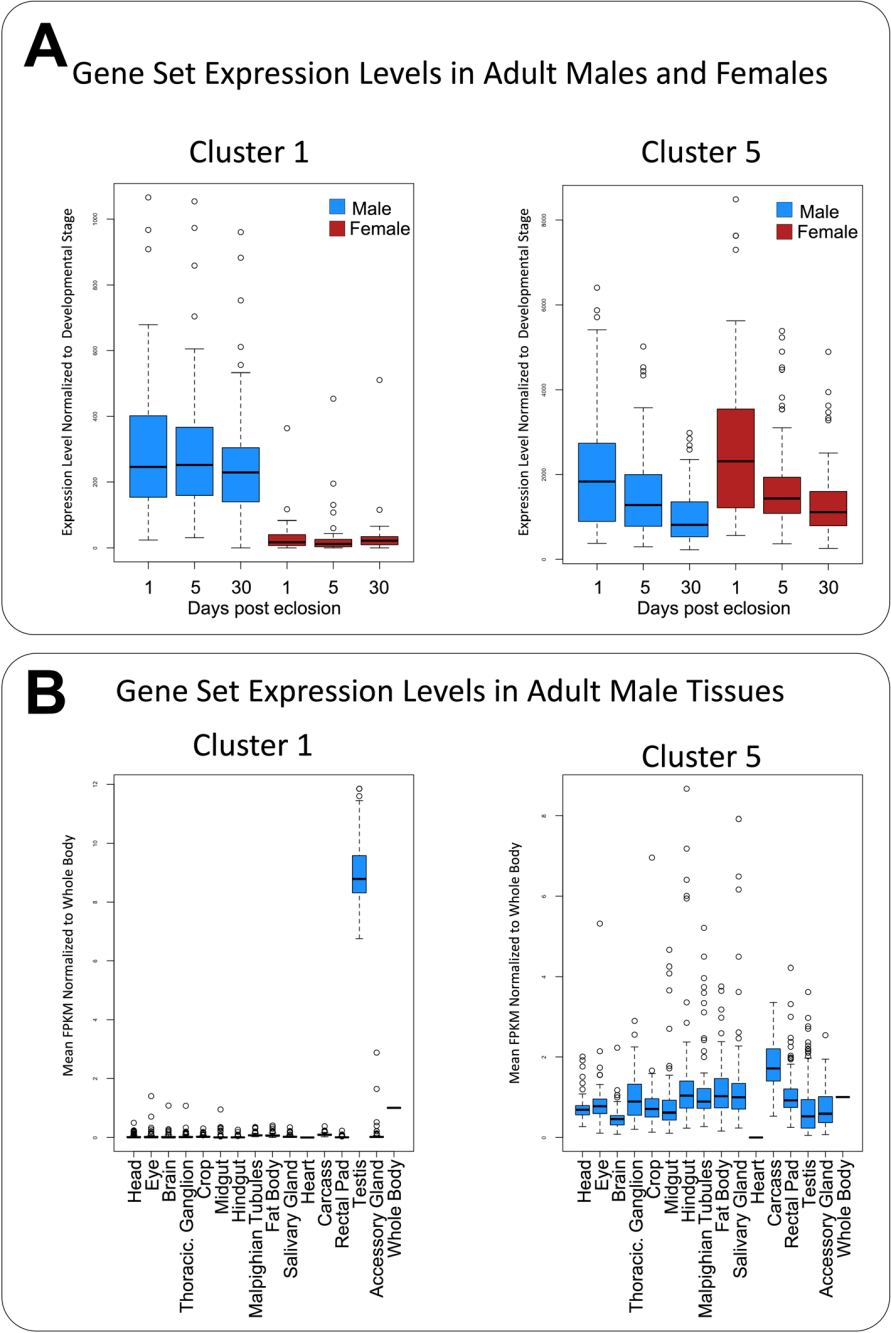
Developmental stage-specific and tissue-specific expression for genes in different co-expressed clusters. Genes identified as having differential expression between mitonuclear genotype in response to refeeding with rapamycin from the cluster 1 and cluster 5 expression profiles were independently analyzed for enrichment of expression at different developmental stages or in different tissues. **a** The *Drosophila* modEncode Developmental Transcriptome Profile data set [40] was used to examine development stage specific gene expression. Expression levels provided by the public data set for genes in cluster 1 (left) and cluster 5 (right) were normalized to the mean level of all genes measured at the specified developmental stage. Displayed are the normalized expression levels observed for male (blue) and female (red) samples at 1, 5 and 30 days post eclosion. **b** The FlyAtlas2 database [41] was used to examine tissue specific gene expression patterns. Expression levels for the cluster 1 (left) and cluster 5 (right) genes were collected for each of the 15 different tissues measured in the public dataset, and normalized to the level observed in the whole body sample. Displayed are the normalized FPKM expression levels for each of the 15 male tissues investigated

The detection of multiple genes that map to the same KEGG pathway node led us to the observation that many of the OXPHOS genes in cluster 1 were paralogs of genes in cluster 5. Further investigation revealed that the paralogs in cluster 1 were the predicted results of gene duplication events from the genes in cluster 5. Previous studies have demonstrated an association of young gene duplications, particularly duplication of OXPHOS genes, with testis specific expression patterns [38, 39]. Based on this association, we examined the gene sets from cluster 1 and cluster 5 for development stage-specific and tissue-specific expression patterns. We utilized the modEncode Developmental Transcriptome Profile data set [40] to analyze gene sets for expression patterns across 30 different developmental stages. Our analysis of the genes from cluster 1 revealed a distinct difference in expression levels between adult males and females, but this sexually dimorphic expression pattern was not detected in the genes from cluster 5 (Fig. 5a).

To determine if the gene sets from cluster 1 and 5 had tissue specific transcriptional regulation patterns, we used the FlyAtlas2 database to assess tissue specific enrichment of gene expression [41]. We observed a higher median enrichment of expression in the testes than all other tissues tested for the genes in cluster 1. This trend was not observed for the genes in cluster 5 (Fig. 5b). Together, these data suggested that the expression profile observed for cluster 1 corresponded to a male specific set of genes with enriched expression in the testes.

### Mitonuclear genotype differentially alters tissue specific regulation of distinct functional pathways

**Fig. 6:**
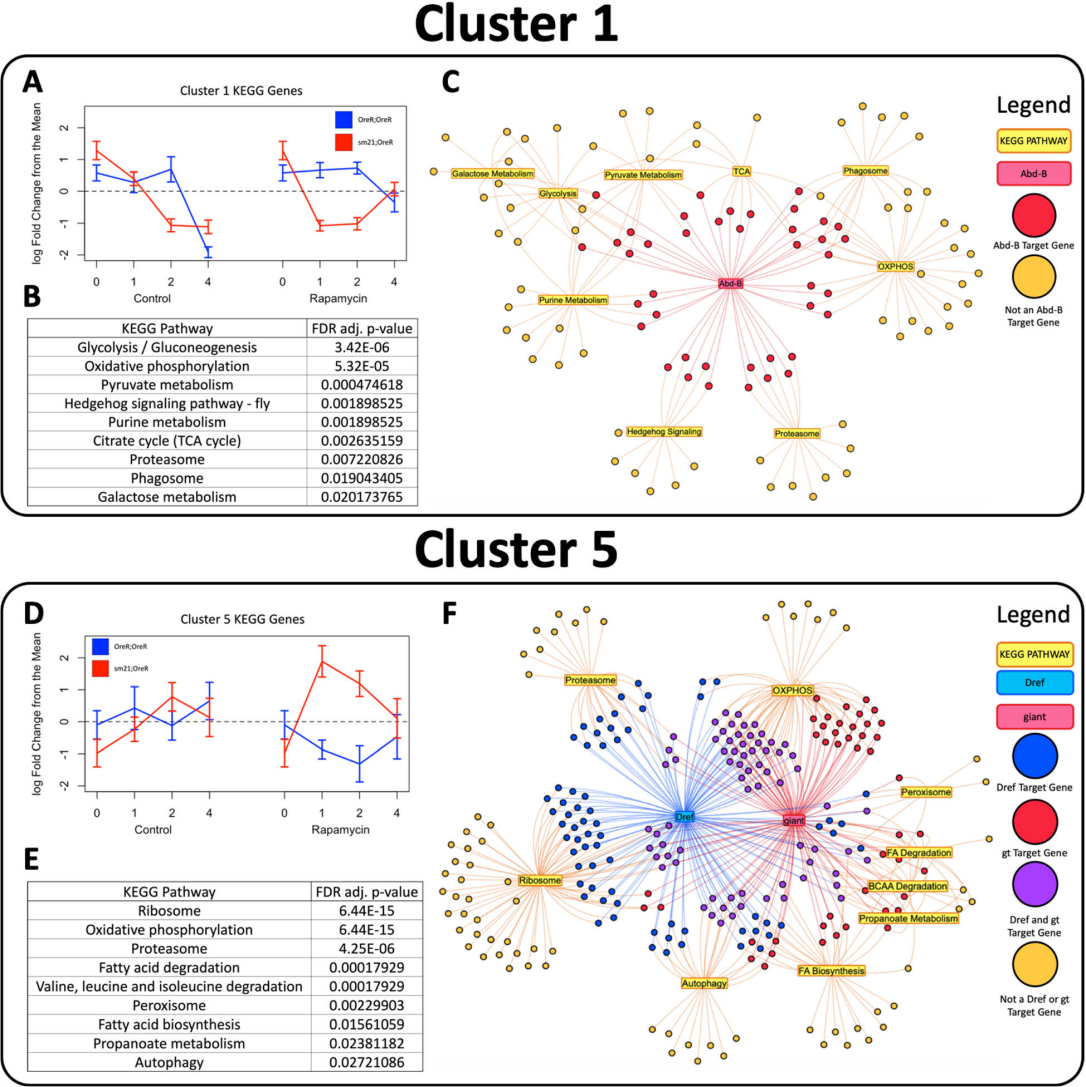
Mitonuclear genotype differentially alters expression of genes associated with distinct functional pathways and transcription factors. KEGG pathway enrichment was performed using the R package GOseq [36] on the gene set from the cluster 1 (top) and cluster 5 (bottom) expression profiles. Expression levels for genes in cluster 1 (**a**) or cluster 5 (**d**) that are associated with the significantly enriched KEGG categories for that cluster were standardized to have zero mean and standard deviation of 1 across all samples. The mean of the standardized expression levels for all genes at each time point were plotted in blue for OreR;OreR samples and red for sm21;OreR samples with control time points plotted on the left and rapamycin treated samples on the right. Error bars are standard deviation among replicate libraries. **b**, **e** Significantly over-represented KEGG categories in the cluster 1 (**b**) and cluster 5 (**e**) gene sets are displayed in the Tables. **c**, **f** Network diagrams generated using the R package visNetwork [42] showing the relationship between enriched transcription factors and KEGG categories for cluster 1 (**c**) and cluster 5 (**f**). **c** Genes from cluster 1 enriched KEGG categories that are associated with the transcription factor *Abd-B* (red) or not (yellow). **f** Genes from cluster 5 enriched KEGG categories that are associated with the transcription factor *Dref* (blue), *giant* (red) or neither (yellow)

Due to the distinct differences between cluster 1 and cluster 5 expression profiles, we performed functional analyses on these two co-expression gene sets separately. KEGG enrichment analysis was done using the supervised set of differentially expressed genes from cluster 1 or cluster 5 independently in order to address the dichotomous impact of mitonuclear genotype on these transcription profiles. There were nine KEGG categories significantly over-represented in the cluster 1 gene set whose function could be broadly characterized as carbohydrate-mediated metabolism and spermatogenesis (Fig. 6a, b). The genes from cluster 1 also showed significant enrichment for sperm associated gene ontology categories with the most over-represented category being “male gamete generation” as detected by GOseq analysis (Supplementary Table S5E). The gene set associated with the cluster 5 expression profile was also enriched for nine KEGG categories. Unlike the functions associated with cluster 1, these categories corresponded with metabolism of proteins and lipids, but were also highly enriched for OXPHOS and autophagy genes (Fig. 6d, e).

Together, these analyses showed associated distinct functional categories with two co-expressed gene sets that had inverse mitonuclear sensitive transcriptional response to rapamycin treatment. In order to test if these differences could be correlated with specific transcriptional regulators, we performed transcription factor enrichment analysis using the R-package RCisTarget [42]. Our analysis found several overrepresented transcription factor binding motifs in the genes from both cluster 1 and cluster 5. The cluster 1 gene set was enriched for the hox gene *Abdominal B* (*Abd-B*) (Supplementary Table S6A) which has enriched expression in the testis, consistent with the testis specific gene expression detected for these co-expressed genes. These *Abd-B* associated genes were found in all nine cluster 1 enriched KEGG categories (Fig. 6c). The genes in cluster 5 were significantly enriched for motifs associated with 28 different transcription factors (Supplementary Table S6B), none of which were found in the analysis of the cluster 1 gene set. Two of the most highly enriched transcription factors for cluster 5 were *Dref* (DNA replication-related element factor) which has been shown to function in response to mTOR activity and *gt (giant)* which, through previous work from our lab, has been associated with mitonuclear genotype sensitive gene expression [43, 44]. Together, *Dref* and *gt* were associated with 185 of the 258 genes in the enriched KEGG pathways for cluster 5 (Fig. 6f).

## Discussion

In recent years it has become apparent that mitochondria are more than energy producing organelles, playing a diverse role in regulation of cellular functions such as redox regulation, nutrient signaling, protein homeostasis, lipid metabolism, apoptosis, nucleotide biosynthesis, and regulation of chromatin accessibility [45–48]. Altered regulation of these functions has been linked to disorders such as cancer and neurodegenerative diseases [49, 50]. Cellular regulation of mitochondrial OXPHOS activity is largely maintained through metabolite availability and mitochondrial quantity. The TOR signaling pathway has an established role in mediating metabolic homeostasis through regulation of nutrient signaling, protein synthesis, and autophagy [51]. Modulation of TOR signaling has been demonstrated to regulate mitochondrial function and mitochondrial biogenesis [10, 52]. Many studies prioritize focus on TOR kinase activity through post-translational effects; however, several TOR-mediated mitochondrial regulatory mechanisms function through activation of transcription factors [53, 54]. In order to modulate mitochondrial activity, signals relaying mitochondrial status must be communicating with the regulator through retrograde mitochondrial signaling. The mechanisms underlying this communication with TOR signaling, including how TOR mediated transcriptional regulation is coordinated with that of the mtDNA encoded components, are poorly understood.

In this work, we have taken a genetic approach to understanding retrograde signaling by testing metabolic responsiveness in mitochondrial introgression lines to address two hypotheses. Our first hypothesis was that mtDNA genotype would alter transcriptional responsiveness to dietary flux. Our second hypothesis was that mtDNA genotype would alter the transcriptional responsiveness to rapamycin under conditions of dietary flux. Both of these hypotheses address the impact of communication between the mitochondrial and nuclear genomes on critical stress response mechanisms. Our results provide limited support for the first hypothesis, but strong support for the second hypothesis. The impact of alternative mtDNA genotypes on the transcriptional response of components of core metabolic pathways was much more pronounced during nutrient influx under rapamycin treatment than under control conditions.

The impact of alternative mtDNA genotypes on refeeding under control conditions was minimal. In the comparison between the OreR;OreR and sm21;OreR in response to refeeding without rapamycin treatment, there were 215 genes detected to have a differential response pattern across the two time courses. Analysis of these genes revealed no significantly enriched KEGG pathways. On the other hand, the comparison between mtDNA genotypes in response to refeeding with rapamycin revealed over 4000 differentially responding genes that represented several, significantly enriched KEGG pathways. The most highly enriched genes were found in core metabolic pathways that connect the nutrient utilization pathways glycolysis, fatty acid degradation, and protein degradation to the TCA cycle and OXPHOS. The expression profiles for these genes demonstrated distinct regulatory patterns between mtDNA genotypes that converge onto a similar level of expression. This implies a difference in mechanism for reaching homeostasis, presumably a compensation to maximize efficiency.

Our analysis revealed that the genes with different responses to mtDNA genotype had two distinctly different transcriptional profiles contributing to the functionally enriched KEGG categories. Gene sets in expression profile cluster 1 and cluster 5 displayed opposite directionality across the time course between genotypes in response to refeeding plus rapamycin, and were the largest contributors to 15 of the 22 enriched KEGG categories. Many of the genes in these two co-expression clusters were mapped to the same functional KEGG pathway node. For OXPHOS, we found this was due to the presence of gene duplicates. According to the “out of testes” hypothesis, young duplicate genes are prone to have testis-specific expression enrichment due to increased chromatin accessibility during meiosis and the increased evolutionary pressure to use mutated genes [38, 39]. Interestingly, when we analyzed the entire cluster 1 gene set using modEncode and FlyAtlas2 data sets, we found an expression pattern across developmental stages and isolated tissues consistent with testis specific expression. This testis specific expression pattern was found for all of the genes in cluster 1, not just those in OXPHOS and not only those that we identified as gene duplicates. Functional analysis of cluster 1 genes showed that they were significantly enriched for KEGG categories related to carbohydrate-dependent aerobic energy production and spermatogenesis, and for GO terms associated with spermatogenesis. Although we determined that these genes were being co-regulated differently than the cluster 5 genes in a testis-specific manner and were enriched for spermatogenesis associated functions, a precise mechanism was unclear. As previously stated, chromatin accessibility during meiosis is predicted to be a major contributor to testis-specific transcription. Interestingly, it has been demonstrated that rapamycin can inhibit the initiating steps of spermatogenic meiosis [55–57]. Our data for the genes in cluster 1 shows that OreR;OreR has an inhibited transcriptional response to refeeding when treated with rapamycin, but an amplified response in the sm21;OreR line. If transcription of the genes in this cluster are related to a meiotic chromatin state, then the differential expression between genotypes is consistent with rapamycin inhibiting spermatogenesis in OreR;OreR but not sm21;OreR.

While our experiment was not designed to detect transcriptional responsiveness in the testis, the findings from our analysis have several significant implications for further research focused on this testis specific gene set. First, the response observed in control samples suggest testis specific genes are regulated differently in response to refeeding than other tissues. It is important to note that the genes in cluster 5 are also likely expressed in testis, but it is beyond the scope of our experiment to determine if the cluster 5 genes are similarly regulated in the two tissue types. Our observation that the two gene sets are enriched for different transcription factors however indicates that the cluster 1 genes could represent a tissue specific gene expression response to refeeding. Second, this work suggests that these testis specific genes are sensitive to rapamycin treatment in a mitonuclear genotype dependent manner. While mitonuclear genotype alone did not significantly alter gene expression levels during refeeding for the genes in cluster 1 or cluster 5, it did have a significant effect on gene expression during refeeding with rapamycin. Again, our experimental design does not give us the power to determine the response profile of cluster 5 genes in the testis, but the different interaction of the genes in these two clusters is consistent with a different regulatory mechanism. That is, not only do they have a different response to refeeding alone, but the mitonuclear genotype alters the transcription profile in response to rapamycin differently as well (transient decrease in cluster 1 genes and transient increase in cluster 5 genes). Third, we detected a distinct enrichment of OXPHOS genes that are proposed products of duplication events in cluster 1. The testis specific expression of gene duplicates and paralogs has been well established, but many of their functions and regulatory mechanisms are still unclear [38, 39, 58, 59]. Altogether, these findings present novel insight into the regulation of testis specific genes associated spermatogenesis and metabolism pathways by identifying a role for mitonuclear genetic interactions and metabolic stress.

The comparison between genotypes revealed the most significant differences in the rapamycin-treated expression profiles in the earliest time points when the flies were also responding to refeeding after fasting for 12 h. We did not observe a significant difference between genotypes in response to refeeding under control conditions, suggesting the mitonuclear genotype specific response is mediated by the rapamycin treatment and not refeeding. Unlike cluster 1, the genes in cluster 5 were not found to be more highly expressed in testis. This suggests that this gene set better represents the significant differentially responding genes between mitonuclear genotypes in response to rapamycin treatment since they are expressed throughout the majority of somatic tissues assessed. The KEGG functional analysis of cluster 5 genes revealed enrichment for pathways that are considered the key canonical outputs of mTORC1 function including protein metabolism, fatty acid metabolism, autophagy and OXPHOS. Notably, these pathways included all of the baseline response pathways observed in the OreR;OreR response to refeeding with control food indicating the mitonuclear genotype is specifically disrupting the refeeding response. This functional assessment supports our hypothesis that mtDNA genotype alters the transcriptional responsiveness to rapamycin under conditions of dietary flux. However, the transcriptional response profile was unexpected. An anticipated outcome was that rapamycin treatment would inhibit TOR-mediated signaling during refeeding since both rapamycin treatment and nutrient deprivation are known to inhibit TOR activity [51]. This appears to be the case in OreR;OreR, where the altered expression of genes with a significant response to refeeding was delayed or less intense in the presence of rapamycin. However, in the sm21;OreR line, significant upregulation of the expression of these genes was stimulated by refeeding plus rapamycin. This occurred more rapidly than a similar shift in expression that was seen with refeeding under control conditions. The expression levels of these genes culminated in similar levels for both mitonuclear genotypes by the fourth hour of rapamycin treatment. These findings were unexpected for two reasons. First, we did not anticipate that rapamycin treatment would heighten sensitivity to refeeding. Second, we expected a shift in expression levels at the end of the time course. Instead, these genes in the sm21;OreR genotype demonstrated increased responsiveness to nutrient flux in the presence of rapamycin before ultimately displaying a transcriptional response similar to the OreR;OreR rapamycin samples.

These transcriptional data shed light on our earlier metabolomic data comparing these same genotypes exposed to control and rapamycin-containing diets [4]. In that study, the metabolite profiles of control and rapamycin-treated sm21;OreR genotype overlapped in principal component space for carbohydrates and proteins, while the OreR;OreR genotype had distinct metabolite profiles on these two treatments. We interpreted this as a form of metabolic reprogramming induced by the ‘disrupted’ sm21;OreR mitonuclear genotype that was not induced in the ‘native’ mitonuclear genotype [4]. While the association of those metabolite data and the new transcriptional data requires further experimental analyses (the prior study used females treated for 10 days, while the current study used males treated for 4 hours), it is notable that the metabolites showing these effects represented canonical TOR functions, as indicated above.

While we were able to demonstrate a distinct impact of mtDNA genotype on canonical mTORC1 pathways and testis specific genes, we were unable to identify specific causal mechanisms for the observed transcriptional responsiveness. In the gene sets from our major co-expression clusters, cluster 1 and cluster 5, there was a distinct shift in expression at the earliest time point examined post starvation for the sm21;OreR samples when refed with rapamycin. It is puzzling that treatment with an mTOR inhibitor would activate canonical mTORC1 outputs more readily than refeeding alone. One possibility is that the observed shift in responsiveness across pathways is due to differential regulation of specific transcription factors. Our data found that cluster 1 and cluster 5 gene sets were distinctly enriched for transcription factor binding sites. In cluster 1, binding site motifs for the testis expressed transcription factor *Abd-B* was found significantly enriched. *Abd-B* has been shown to play a role in spermatogenesis with higher levels inhibiting germ line stem cell differentiation in an epigenetic dependent manner [60]. In this study, we observed a decrease in transcript level for inferred *Abd-B* targets in response to refeeding with rapamycin for sm21;OreR but not for OreR;OreR. This could indicate that the functional consequences of altered mitonuclear interactions is influencing mTOR’s role in regulating spermatogenesis and that this is due to differences in the epigenetic state.

Two of the transcription factors found enriched in the cluster 5 gene set, *Dref* and *gt*, have particularly conspicuous relevance to our experimental conditions. *Dref* has been shown to function downstream of mTOR and mediate processes associated with nutrient deprivation such as ribosomal biogenesis and lipid metabolism [43]. Since the genes in cluster 5 with proposed *Dref* regulation are upregulated in the sm21;OreR early response to rapamycin but not in OreR;OreR, this suggests that the functional consequences of altered mitonuclear genotype influences mTOR’s regulation of *Dref*. While Killip et al. (2012) studied larval fat body in a *Dref* knockdown model, and our results are from eviscerated abdomen samples of adults with altered mitochondrial genotypes, our shared findings point to *Dref* as an important factor in the coordination between mTOR and mitochondrial function.

The transcription factor *gt* was previously detected in work from our laboratory as a mitonuclear genotype sensitive transcription factor [44]. It is notable that, while this previous study was also conducted to compare the effects of mitonuclear genetic interactions, it was done so using different mitonuclear genotypes than our current study. Together, these findings suggest that *gt* may be a conserved component of the retrograde signaling pathway.

Another possibility is that the difference in transcriptional responsiveness is due to epigenetic modification and chromatin remodeling. Increased DNA accessibility could explain the observed early transcriptional response to refeeding with rapamycin. Recent studies have implicated reactive oxygen species, NAD+/NADH ratios, ATP/ADP ratios, acetyl CoA availability and components of the mitochondrial unfolded protein response (UPRmt) as likely sources of mitochondrial signals that alter nuclear transcriptional states through epigenetic modifications and chromatin remodeling [45, 61–63]. However, none of these studies have used alternative mtDNAs in their analyses. Our analysis of differentially expressed genes between mitonuclear genotypes revealed OXPHOS to be one of the most significantly enriched pathways, which, if functionally altered, could contribute to differences in these mitochondrial signals that induce epigenetic modifications. Histone modifications and the UPRmt have also been shown to be activated by mitochondrial dysfunction factors such mtDNA mutation, the mitochondrial stress response pathway and altered proteostasis [47, 64, 65]. While our model is not utilizing a dysfunctional mtDNA, it could still potentially be detected as such due to altered interactions with the nuclear genome. Taken together, we interpret our results as indicating that mitonuclear genotype affects metabolic factors that are known to modulate the epigenetic state, possibly explaining the rapamycin-mediated increase in transcriptional responsiveness for the sm21;OreR genotype.

## Conclusions

In summary, we have demonstrated that the presence of an alternative mtDNA can induce a large-scale disruption of gene expression. We consider it likely that this is accounted for by treatment with rapamycin since refeeding alone resulted in similar shifts in expression between genotypes. Our analysis of the temporal response to refeeding revealed distinct response patterns that would have been otherwise undetectable in single time point comparisons. The effects of rapamycin on gene expression were observed as a transient differential shift between genotypes followed by a convergence to similar levels at the final time point. Because of this difference between genotypes at each refeeding time point, these patterns would have not only gone undetected, but could have been misleading. Detection of this temporal response revealed an important role for mitonuclear communication in achieving homeostasis through canonical mTORC1 mediated signaling pathways. It also allowed us to uncover an impact of mitonuclear genotype by identifying testis specific genes with differential expression between the two genotypes. While it will be important to examine additional combinations of nuclear and mitochondrial genotypes, our findings provide a new context for future work on mitochondrial retrograde signaling and its relationship to TOR signaling.

## Methods

### Overview of experimental design

To investigate the effect of mtDNA genotype on the transcriptional response to metabolic stress, we performed transcriptome analysis on eviscerated abdomens. We used males from the Oregon R *Drosophila* line and also the mitochondrial introgression line that has a sm21 mtDNA haplotype and an Oregon R isogenic nuclear genome. This mitochondrial introgression line was developed as described in [21]. The *Drosophila* strains referred to using the mtDNA;nucDNA notation, with the Oregon R line referred to as OreR;OreR and the introgression line referred to as sm21;OreR. We chose to focus on the eviscerated abdomen due to its enrichment of tissues with innate responsiveness to variations in nutritional state such as muscle and fat body. The experimental metabolic stress was designed to capture the effect of mtDNA genotype on the transcriptional response to drastic metabolic variation (Fig. 1a). OreR;OreR and sm21;OreR flies were starved overnight to induce a metabolically stressed state and to inhibit mTORC1 activity. The 12 h starved state is the starting condition for our time course. Post starvation, flies were treated for 30 min with 200 μM rapamycin or ethanol vehicle but no food. After the 30 min treatment period, the flies were given access to lab food containing rapamycin or ethanol vehicle. Flies were collected at 0 (12 h fasting), 1 (30 min agar + treatment followed by 30 min food + treatment), 2 and 4 h post starvation for transcriptome analysis. To summarize, the time course treatment was initiated with 21 vials of 30 adult males of each genotype for a total of 42 vials. At each time point, three vials of flies were frozen for each genotype and treatment. Each genotype had only a single time point 0 condition (denote condition) that shared for each rapamycin time course for that genotype. Thus, the design is: 2 genotypes × 2 treatments (vehicle or rapamycin) × 3 timepoints × 3 replicates = 36 post starvation samples, plus 3 time point 0 samples (denote condition) for each genotype, for a total for 42 independent samples. Transcriptome analysis was then conducted on 10 eviscerated abdomens collected from each sample for a total of 420 dissected flies.

### Drosophila stocks

We utilized the two fly stocks OreR;OreR and sm21;OreR with common nuclear genomes and different mtDNA haplotypes (notation is as follows: mtDNA;nuclearDNA). These stocks were generated using balancer chromosome replacement crosses where the *D. melanogaster* OregonR (OreR) chromosomes were placed onto distinct cytoplasmic backgrounds. This was achieved using crosses where the female cytoplasm was derived from lines carrying different mtDNAs, and the nuclear chromosomes were introduced through the male parent. Details of this process are described in Montooth et al. 2010. The sm21;OreR introgression line has been regularly backcrossed with the control OreR;OreR line in an effort to maintain an isogenic nuclear genome. This is done by crossing virgin introgression females with Oregon-R control males for several generations. Flies were maintained on a 12 h light-dark cycle on our standard lab diet containing 5.2% cornmeal, 2% yeast, 11% sugar and .9% agar. Egg lays were conducted with 20 mating pairs of age matched adults for 48 h. Progeny are collected 12 days after the egg lay and maintained on lab food until they reach 5 days post-eclosion at which point they are considered adults.

### Refeeding scheme

Adult flies were starved overnight for 12 h on 2% agar before being refed with or without rapamycin. This was done using 5–6 day post-eclosion mated male flies from the Oregon R control genotype and sm21;Oregon R introgression lines maintained on standard lab diet. Cohorts of 30 flies were transferred to starvation diet vials (2% Bacto agar prepared with distilled water) overnight for 12 h. They were then transferred to agar vials with 200 μM rapamycin or ethanol vehicle for 30 min followed by transfer to standard lab fly food containing 200 μM rapamycin or ethanol vehicle. A cohort from each genotype was collected and flash frozen at 4 different time points. The initial “starved” time point was collected just before refeeding followed by collecting of samples at 1, 2 and 4-h post-starvation (Fig. 1a). This was done for both genotypes in control and rapamycin treatments for a total of 14 different conditions. The flies were flash frozen and stored at -80C.

### Western blot

Protein was extracted from frozen whole flies using 400ul lysis buffer (10 mM Tris-base, pH 7.6, 5 mM EDTA, 50 mM sodium chloride, 30 mM sodium pyrophosphate, 50 mM sodium fluoride and 100uM sodium orthovanadate,10μg/ml aprotinin, 10μg/ml leupeptin, 0.14 mM AEBSF, 1μg/ml microcystin, and 1% Triton-100) per 30 flies. Protein concentrations were measured using the Pierce BCA Protein Assay (Thermo Scientific Franklin, MA). Equal amounts of protein (40μg) was loaded onto 10% SDS-PAGE gels. Gels were transferred to PVDF membranes, blocked in 5% milk for 1 h at 37 °C and incubated overnight with primary antibody to phospho-*Drosophila* p70 S6 Kinase (Thr398) (Cell Signaling #9209, Danvers, MA). ECL Prime (GE Healthcare, Marlborough, MA) was used to develop the blots. Multiple exposures were acquired using the ChemiDoc-It imaging system (UVP, Upland, CA).

### Total RNA extraction and sequencing

For each of the 42 samples, total RNA was extracted from 10 eviscerated abdomens using a phenol-chloroform extraction followed by ethanol precipitation. A total of 420 male flies were dissected on an ice-cold dissection block removing the head, thorax and loose components of the abdomen. The eviscerated abdomens were then placed in chilled TRIzol and homogenized at 30hz for 4 min in a TissueLyser®. Total RNA was extracted from cell lysates with chloroform using phase lock gel tubes (VWR, Rednor, PA) followed by alcohol precipitation and resuspension in molecular biology grade water. DNA was removed using a Turbo DNA free kit and a final cleanup was done using a Zymo clean and concentrator-5 kit. Concentration and contamination was assessed by nanodrop analysis with additional quality control steps performed by Genewiz, Inc. (South Plainfield, NJ). Transcriptome sequencing was done by Genewiz using an Illumina HiSeq2500 at 6 samples per lane with 50 base pair single end reads.

### Transcriptome analysis

Quality control of each transcriptome library was assessed using the program FastQC version 0.10.1 [66]. The reads were aligned to the *D. melanogaster* BDGP R5/dm3 genome assembly using Tophat version 2.1.1 [67]. The BAM files containing the read alignment data were compiled into read count tables using the program HTSeq [68]. Genetic quality control for the isogenic backgrounds was manually examined with the Integrative Genomic Viewer (IGV) to detect single nucleotide polymorphism differences between the two mtDNA genotype nuclear backgrounds [69–71]. We analyzed the libraries using an MDS distance matrix to quantify the level of similarity between replicates and identify outlier libraries. This revealed several outliers resulting in the exclusion of a single replicate library from each genotype x treatment x time point condition. This resulted in 2 libraries per time point × 4 time points = 8 libraries for each experimental time course. Comparative analysis between transcriptomes of individual conditions at specific time points was performed using the R package EdgeR [30]. Additionally, we used the R package ImpulseDE2 [31] which utilizes DESeq2 modeling to test longitudinal data sets for differential expression trajectories. The significance cutoff for differential gene expression with EdgeR or ImpulseDE2 was set to a Benjamini-Hochberg false-discovery rate corrected *p*-value of 0.05. Differentially expressed genes were subjected to model based clustering with the R package MBCluser.Seq [32] to segregate them into similar expression trajectory profiles. Gene ontology enrichment analysis was done using the R package GOseq [36]. Pathway visualization using native KEGG html files was performed using the KEGG mapper function provided by Kanehisa Laboratories (https://www.kegg.jp/).

### RT-qPCR

Total RNA was prepared following the same protocol used for transcriptome sequencing on eviscerated abdomens from sm21;OreR samples refed with rapamycin treated food for 1 or 4 h after fasting. RNA was reverse transcribed using a Maxima H Minus First Strand cDNA Synthesis Kit (Thermo Scientific Franklin, MA). The qPCR reaction was performed using PowerUp SYBR Green Master Mix (Thermo Scientific Franklin, MA). Gene targets, primer details and qPCR results are listed in Supplementary Table S7A-B.

### Development stage and tissue specific expression analysis

Gene expression enrichment across developmental stages was analyzed using the publicly available modEncode Developmental Transcriptome Profile data set [40]. For a given gene, the FPKM expression level at each developmental stage was normalized to the total gene expression at that stage. The data was downloaded from https://github.com/modENCODE-DCC/www/tree/master/html/docs/flyscores.

The tissue specific gene expression analysis was generated using the publicly available FlyAtlas2 database [41]. Gene expression collected from tissue specific RNA-seq analyses was normalized to the level observed in whole fly samples for each gene of interest. The FlyAtlas2 data set was downloaded from motif.gla.ac.uk/downloads/FlyAtlas2_19.10.15.sql.

### Transcription factor binding motif analysis

Enrichment of transcription factor binding motifs within our gene sets was generated using the R package Rcis Target [42]. The motif to annotation database v8 was downloaded from https://resources.aertslab.org/cistarget/motif2tf/motifs-v8-nr.flybase-m0.001-o0.0.tbl and the motif ranking database was downloaded from https://resources.aertslab.org/cistarget/databases/drosophila_melanogaster/dm6/flybase_r6.02/mc8nr/gene_based/dm6-5kb-upstream-full-tx-11species.mc8nr.feather. Transcription factors with a normalized enrichment score > 3 were considered significantly enriched.

## Supplementary Information

**Additional file 1: Table S1.** Count table. Read count data before normalization.

**Additional file 2: Table S2.** (A-L). Individual time point differential expression analysis. Output files from the edgeR analysis of individual contrasts between refed conditions and starved state. The file contains the specific contrast in the tab name.

**Additional file 3: Table S3.** (A-H). Longitudinal time course differential expression analysis. ImpulseDE2 analysis output for individual time courses and comparisons between time courses. The file has the time course condition(s) that were analyzed denoted in the tab name.

**Additional file 4: Table S4.** MBCluster.seq heatmap data and cluster assignment. Output file from the model-based clustering generated by MBCluster.seq including the relative expression used for the heatmap and cluster assignment for individual genes.

**Additional file 5: Table S5.** Gene ontology and KEGG pathway enrichment analysis. Output file from the GO term and KEGG pathway enrichment analysis for specified gene sets. The file has the output files for the following analyses in tabs A-H: (A-B) Output file from the GO term (A) and KEGG pathway (B) enrichment analysis of genes found significantly different response profiles between the OreR;OreR and sm21;OreR control treatment time courses. (C-D) Output file from the GO term (C) and KEGG pathway (D) enrichment analysis of genes found significantly different response profiles between the OreR;OreR and sm21;OreR Rapamycin treatment time courses. (E-F) Output file from the GO term (E) and KEGG pathway (F) enrichment analysis of genes found in the cluster 1 gene set. (G-H) Output file from the GO term (G) and KEGG pathway (H) enrichment analysis of genes found in the cluster 5 gene set.

**Additional file 6: Table S6.** (A-B). Transcription factor binding motif analysis. Output file for genes in cluster 1 (A) or cluster 5 (B) from Rcis-Target that includes the enrichment score data for transcription factors and the associated genes.

**Additional file 7: Table S7.** RT-qPCR Results. (A) Table with gene targets and primer sequences used. (B) Table of Ct values for genes used in the time course validation. Transcript abundance was measured for four genes that displayed a significant difference in expression between the sm21;OreR samples treated for 1 hour with rapamycin and those treated for 4 hours. The table contains the gene symbol, flybase gene id, sample condition, biological replicate number, Ct (mean of technical replicates) and the read counts per million detected for the transcript in the RNAseq experiment.

**Additional file 8: Figure S1.** Western blot analysis. The raw western blot images of three biological replicates (replicate 1 on left, replicate 2 center and replicate 3 on right) probed for phosphorylated S6K1 (top) and then probed for B-Actin (bottom) as an additional loading control. Each biological replicate included all conditions used in the RNAseq experiment. In addition, there are rapamycin treated and untreated non-fasted positive controls for each genotype. The labels for sample conditions indicate the refeeding duration (0, 1, 2 or 4 hours of refeeding or non-fasted positive control), the food treatment type (control food (C) or rapamycin treated food (R)) and the genotype (OreR;OreR (O) or sm21;OreR (S)).

**Additional file 9: Figure S2.** Volcano plots of individual time point differential expression analysis relative to the fasted state. Volcano plots visualizing the analysis of differential expression for each post-refeeding condition relative to the time 0 starved state was performed using edgeR. Log fold change in expression from time 0 is plotted on the x-axis and the -log10 FDR is on the y-axis. Genes with significant differential expression (FDR adjusted *p*-value < 0.05, red trendline) are colored red and all others black.

**Additional file 10: Figure S3.** Volcano plots of inter-genotype differential expression analysis. Volcano plots visualizing the analysis of differential expression between genotypes at single time point x treatment conditions (top, transient response) or combined time point comparisons (bottom, sustained response). (A) Total number of differentially expressed genes in the transient response analysis detected by EdgeR (Control refeeding in red and Rapamycin refeeding in blue). (B-H) Volcano plots of the EdgeR results from the transient response analysis. Log fold change in expression from time 0 is plotted on the x-axis and the -log10 FDR is on the y-axis. Genes with significant differential expression (FDR adjusted p-value < 0.05, red trendline) are colored red and all others black. Treatment x time point conditions being compared in each volcano plot: (B) sm21;OreR fasted vs OreR;OreR fasted (C) sm21;OreR 1 hour control diet vs OreR;OreR 1 hour control diet (D) sm21;OreR 2 hour control diet vs OreR; OreR 2 hour control diet (E) sm21;OreR 4 hour control diet vs OreR;OreR 4 hour control diet (F) sm21;OreR 1 hour rapamycin diet vs OreR;OreR 1 hour rapamycin diet (G) sm21;OreR 2 hour rapamycin diet vs OreR; OreR 2 hour rapamycin diet (H) sm21;OreR 4 hour rapamycin diet vs OreR; OreR 4 hour rapamycin diet. (I) Total number of differentially expressed genes in the sustained response analysis detected by EdgeR (Control refeeding in red and Rapamycin refeeding in blue). (J-M) Volcano plots of the EdgeR results from the sustained response analysis. Log fold change in expression from time 0 is plotted on the x-axis and the -log10 FDR is on the y-axis. Genes with significant differential expression (FDR adjusted p-value < 0.05, red trendline) are colored red and all others black. Treatment x time point conditions being compared in each volcano plot: (J) sm21;OreR 1 and 2 hour control diet vs OreR;OreR 1 and 2 hour control diet (K) sm21;OreR 2 and 4 hour control diet vs OreR;OreR 2 and 4 hour control diet (L) sm21;OreR 1 and 2 hour rapamycin diet vs OreR;OreR 1 and 2 hour rapamycin diet (M) sm21;OreR 2 and 4 hour rapamycin diet vs OreR;OreR 2 and 4 hour rapamycin diet.

**Additional file 11: Figure S4.** (A-U). Heatmaps of gene expression in enriched KEGG pathways. Each of the 22 KEGG categories enriched in the genes found to have significantly different response patterns to refeeding with rapamycin treatment between the OreR;OreR and sm21;OreR mtDNA genotypes. The x-axis is the genotype by treatment condition for each time point. The y-axis shows the genes in the KEGG category that were found to have significantly different expression between genotypes when treated with rapamycin on the right and the colors on the left denote the expression profile cluster for the gene. Gene expression is displayed as the row normalized z-score.

## Data Availability

The raw RNA-seq reads generated in this study are available from the NCBI Sequence Read Archive (SRA) (https://www.ncbi.nlm.nih.gov/sra) under BioProject accession: PRJNA610872. The dm3 genome assembly is freely available through the Ensembl genome browser at https://oct2014.archive.ensembl.org/Drosophila_melanogaster/Info/Index or directly via ftp at ftp://ftp.ensembl.org/pub/release-77/fasta/drosophila_melanogaster/dna [72]. The modEncode Developmental Transcriptome Profile data set is publicly available at https://github.com/modENCODE-DCC/www/tree/master/html/docs/flyscores [40]. The FlyAtlas2 database with gene expression data collected from tissue specific RNA-seq analyses was downloaded from motif.gla.ac.uk/downloads/FlyAtlas2_19.10.15.sql [41]. The motif to annotation database and the motif ranking database used in the R-cis target transcription factor binding motifs enrichment analysis can be downloaded from https://resources.aertslab.org/cistarget/motif2tf/motifs-v8-nr.flybase-m0.001-o0.0.tbl and https://resources.aertslab.org/cistarget/databases/drosophila_melanogaster/dm6/flybase_r6.02/mc8nr/gene_based/dm6-5kb-upstream-full-tx-11species.mc8nr.feather respectively [42]. R scripts used in the analyses are available upon request, and are posted at https://github.com/DavidRandLab/Santiago-et-al-2021-BMC-Genomics.

## Abbreviations

OXPHOS: Oxidative phosphorylation
mtDNA: mitochondrial DNA
TOR: Target of rapamycin
GxT: Genotype by treatment
GxE: Genotype by environment
KEGG: Kyoto Encyclopedia of Genes and Genomes
GO: Gene ontology
