# Supplementary figures and images for: Mitochondrial genotype alters the impact of rapamycin on the transcriptional response to nutrients in Drosophila
Source: BMC Genomics. 2021 Mar 24;22:213. doi: 10.1186/s12864-021-07516-2 (PMC7992956; doi:10.1186/s12864-021-07516-2)

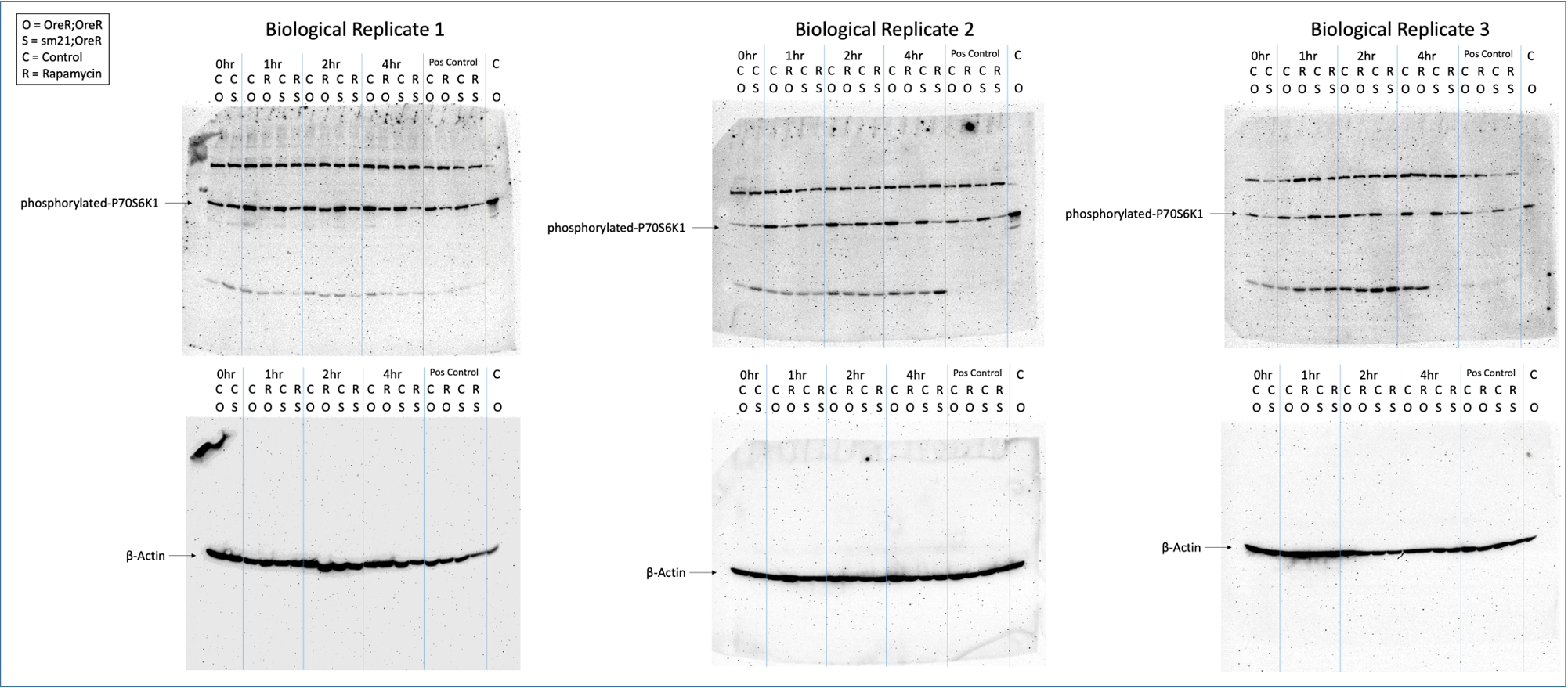

Supplement: Supplementary file 8 — Additional file 8: Figure S1. Western blot analysis. The raw western blot images of three biological replicates (replicate 1 on left, replicate 2 center and replicate 3 on right) probed for phosphorylated S6K1 (top) and then probed for B-Actin (bottom) as an additional loading control. Each biological replicate included all conditions used in the RNAseq experiment. In addition, there are rapamycin treated and untreated non-fasted positive controls for each genotype. The labels for sample conditions indicate the refeeding duration (0, 1, 2 or 4 hours of refeeding or non-fasted positive control), the food treatment type (control food (C) or rapamycin treated food (R)) and the genotype (OreR;OreR (O) or sm21;OreR (S)). [file 12864_2021_7516_MOESM8_ESM.png]

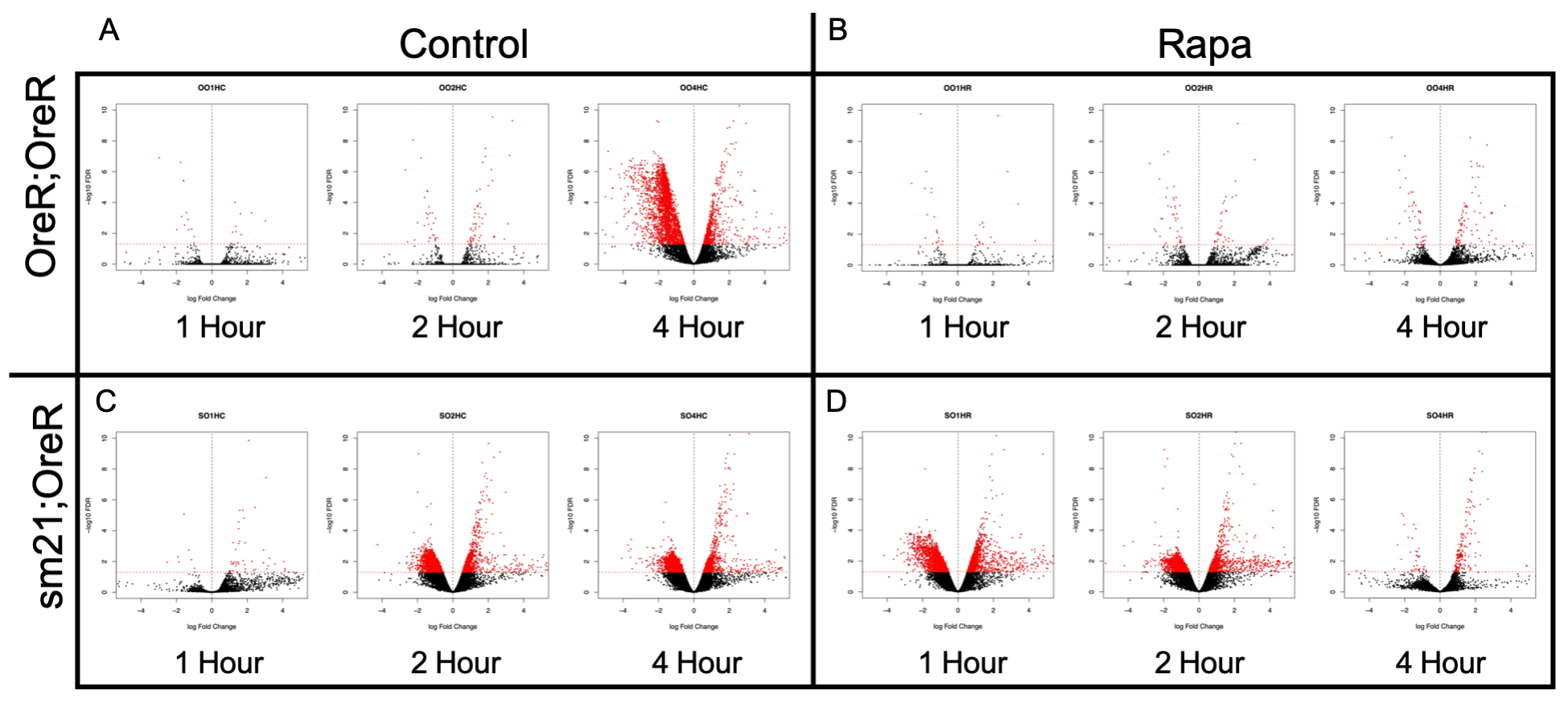

Supplement: Supplementary file 9 — Additional file 9: Figure S2. Volcano plots of individual time point differential expression analysis relative to the fasted state. Volcano plots visualizing the analysis of differential expression for each post-refeeding condition relative to the time 0 starved state was performed using edgeR. Log fold change in expression from time 0 is plotted on the x-axis and the -log10 FDR is on the y-axis. Genes with significant differential expression (FDR adjusted p-value < 0.05, red trendline) are colored red and all others black. [file 12864_2021_7516_MOESM9_ESM.png]

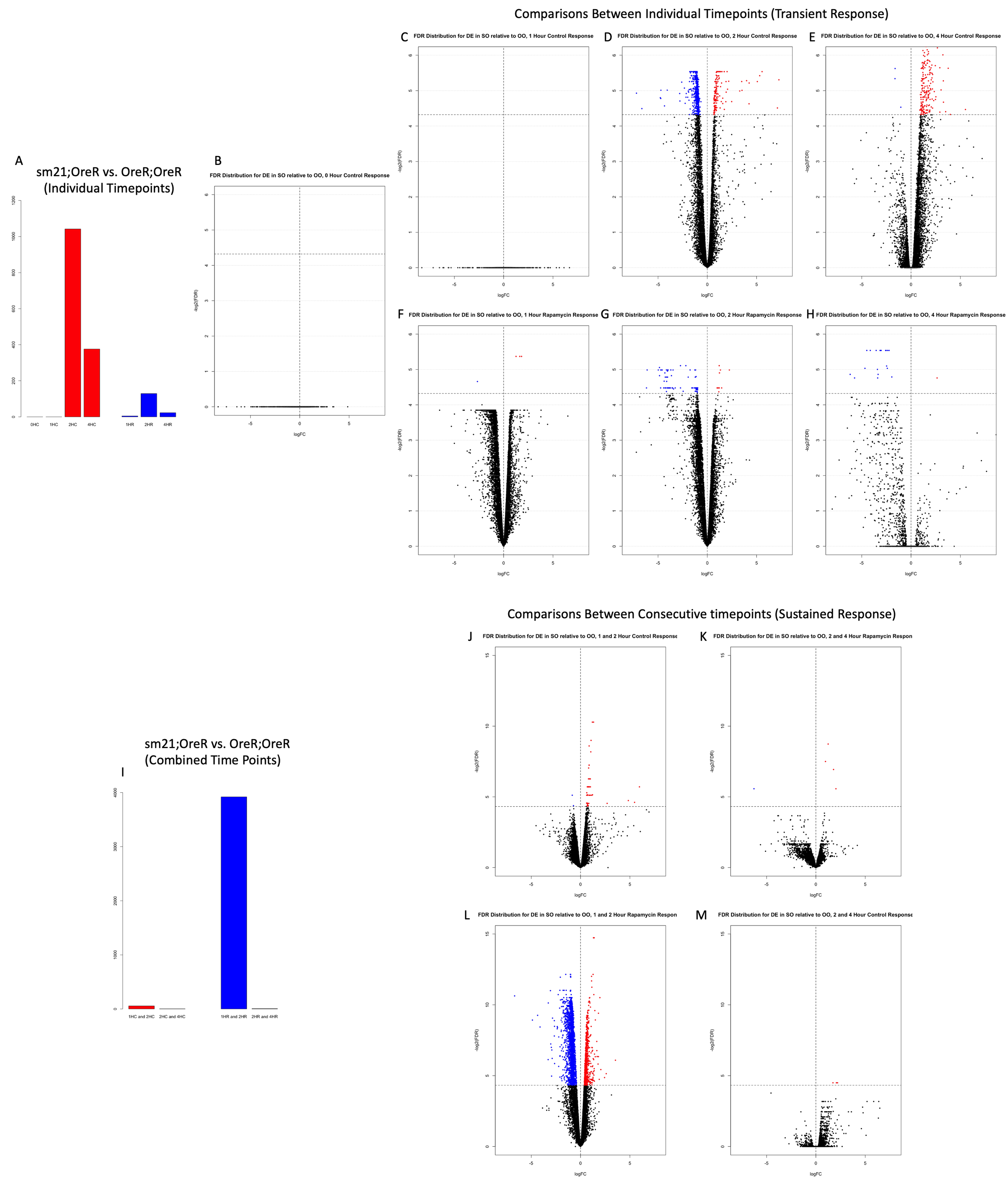

Supplement: Supplementary file 10 — Additional file 10: Figure S3. Volcano plots of inter-genotype differential expression analysis. Volcano plots visualizing the analysis of differential expression between genotypes at single time point x treatment conditions (top, transient response) or combined time point comparisons (bottom, sustained response). (A) Total number of differentially expressed genes in the transient response analysis detected by EdgeR (Control refeeding in red and Rapamycin refeeding in blue). (B-H) Volcano plots of the EdgeR results from the transient response analysis. Log fold change in expression from time 0 is plotted on the x-axis and the -log10 FDR is on the y-axis. Genes with significant differential expression (FDR adjusted p-value < 0.05, red trendline) are colored red and all others black. Treatment x time point conditions being compared in each volcano plot: (B) sm21;OreR fasted vs OreR;OreR fasted (C) sm21;OreR 1 hour control diet vs OreR;OreR 1 hour control diet (D) sm21;OreR 2 hour control diet vs OreR; OreR 2 hour control diet (E) sm21;OreR 4 hour control diet vs OreR;OreR 4 hour control diet (F) sm21;OreR 1 hour rapamycin diet vs OreR;OreR 1 hour rapamycin diet (G) sm21;OreR 2 hour rapamycin diet vs OreR; OreR 2 hour rapamycin diet (H) sm21;OreR 4 hour rapamycin diet vs OreR; OreR 4 hour rapamycin diet. (I) Total number of differentially expressed genes in the sustained response analysis detected by EdgeR (Control refeeding in red and Rapamycin refeeding in blue). (J-M) Volcano plots of the EdgeR results from the sustained response analysis. Log fold change in expression from time 0 is plotted on the x-axis and the -log10 FDR is on the y-axis. Genes with significant differential expression (FDR adjusted p-value < 0.05, red trendline) are colored red and all others black. Treatment x time point conditions being compared in each volcano plot: (J) sm21;OreR 1 and 2 hour control diet vs OreR;OreR 1 and 2 hour control diet (K) sm21;OreR 2 and 4 hour control diet vs OreR;Or [file 12864_2021_7516_MOESM10_ESM.png]
